# Optimising the acceptability and feasibility of novel complex interventions: an iterative, person-based approach to developing the UK Morita therapy outpatient protocol

**DOI:** 10.1186/s40814-017-0181-4

**Published:** 2017-10-03

**Authors:** Holly Victoria Rose Sugg, David A. Richards, Julia Frost

**Affiliations:** Complex Interventions Research Group, University of Exeter Medical School, University of Exeter, Room 1.33 South Cloisters, St Luke’s Campus, Exeter, EX1 2LU UK

**Keywords:** Intervention development, Qualitative research, Person-based approach, Morita therapy, Feasibility study, Depression, Mental health, Protocol

## Abstract

**Background:**

The aim of this paper is to showcase best practice in intervention development by illustrating a systematic, iterative, person-based approach to optimising intervention acceptability and feasibility, as applied to the cross-cultural adaptation of Morita therapy for depression and anxiety.

**Methods:**

We developed the UK Morita therapy outpatient protocol over four stages integrating literature synthesis and qualitative research. Firstly, we conducted in-depth interviews combining qualitative and cognitive interviewing techniques, utilising vignettes of Morita therapy being delivered and analysed using Framework analysis to investigate potential patients and therapists’ perceptions of Morita therapy. Secondly, we developed qualitative themes into recommendations for optimising Morita therapy and synthesised Morita therapy literature in line with these to develop a draft protocol. Thirdly, we conducted repeat interviews with therapists to investigate their views of the protocol. Finally, we responded to these qualitative themes through protocol modification and tailoring our therapist training programme.

**Results:**

As a consequence of literature describing Morita therapy and participants’ perceptions of the approach, we developed both a therapy protocol and therapist training programme which were fit for purpose in proceeding to a UK-based Morita therapy feasibility study. As per our key qualitative findings and resulting recommendations, we structured our protocol according to the four-phased model of Morita therapy, included detailed guidance and warning points, and supported therapists in managing patients’ expectations of the approach.

**Conclusions:**

Our systematic approach towards optimising intervention acceptability and feasibility prioritises the perspectives of those who will deliver and receive the intervention. Thus, we both showcase best practice in intervention development and demonstrate the application of this process to the careful cross-cultural adaptation of an intervention in which balancing both optimisation of and adherence to the approach are key. This presentation of a generalisable process in a transparent and replicable manner will be of interest to those both developing and evaluating complex interventions in the future.

**Electronic supplementary material:**

The online version of this article (10.1186/s40814-017-0181-4) contains supplementary material, which is available to authorized users.

## Background

Clinical depression and generalised anxiety disorder (GAD) are the two most common mental health disorders [1], with one in six people in the UK experiencing such a disorder each year [2]. Many patients are refractory to available interventions [3] such as medication and cognitive behavioural therapy (CBT), with depression and anxiety remaining chronic disorders [1]. Thus, it is important to develop and test new treatments in order to treat a wider range of patients [4] and provide patients with choice alternatives.

### Morita therapy

Morita therapy [5] was developed in Japan in 1919 and originally used in inpatient settings for particular psychological problems, including GAD [6]. The approach is now applied in a variety of ways to a wide range of conditions, including depression, and practiced in countries including North America, Australia, China, Russia and Rwanda [6].

Morita therapy is a holistic approach aiming to improve everyday functioning rather than targeting specific symptoms [6]. Through conceptualising unpleasant emotions as part of the natural ecology of human experience, Morita therapy seeks to re-orientate patients in the natural world and potentiate their natural healing capacity. Morita therapists help patients to move away from symptom preoccupation and combat, which are considered to exacerbate symptoms and interfere with this natural recovery process [7]. By helping patients to accept symptoms as natural features of human emotion which ebb and flow as a matter of course, Morita therapy is in sharp contrast to the focus of established western approaches on symptom reduction and control. In Morita therapy, patients are taught to live with, rather than be without, unpleasant emotions.

### Morita therapy in the UK: the need for an intervention development process

Morita therapy is little known in the UK: neither empirical investigation nor research exploring stakeholders’ views has been undertaken with this population. In line with the Medical Research Council framework for the development and evaluation of complex interventions [8], the authors are currently undertaking a Morita therapy feasibility study to begin such investigations [9]. However, in the absence of research exploring the cross-cultural transferability of Morita therapy, and in the context of multiple possible methods of operationalisation, prior to such a trial, an intervention development process was required to design a comprehensive UK Morita therapy outpatient protocol.

The purpose of this paper is to illustrate an in-depth, iterative, qualitative approach to intervention development, demonstrating best practice in applying the Medical Research Council framework for developing interventions [8] and reflecting the ‘person-based approach’ [10] to optimising intervention acceptability and feasibility prior to a full feasibility study, as applied to the UK Morita therapy outpatient protocol. By alternating and integrating literature synthesis and qualitative research in the cross-cultural adaptation of Morita therapy, our approach prioritises the perspectives of those who will deliver and receive the intervention, whilst ensuring adherence to its core features. This process was essential to proceeding to the feasibility study with a treatment which is both true to the essence of Morita therapy and appropriate, accessible, understandable and deliverable for the target population, particularly in the context of the aforementioned contrast between Morita therapy and established western approaches.

### Study objective

To develop a deliverable and acceptable Morita therapy outpatient protocol for a UK clinical population.

### Research questions

1. Stage one: What are the views and understandings of potential patients and therapists about Morita therapy?
2. Stage two: What can the English-language literature on Morita therapy contribute to the development of an optimal draft protocol?
3. Stage three: What are therapists’ views of Morita therapy, focusing on operationalisability and the accessibility of the draft protocol?
4. Stage four: How should the protocol be optimised and on what should a therapist training programme focus?

## Methods/design

### Study design

Corresponding to the person-based approach’s intervention development phase [10], we developed the protocol over four stages combining exploratory and explanatory components. Stage one involved in-depth exploratory interviews combining qualitative and cognitive interviewing [11] to investigate participants’ views and understandings of Morita therapy. In stage two, we developed qualitative themes into recommendations for optimising Morita therapy and synthesised Morita therapy literature in line with these to develop a draft protocol. Stage three involved repeat in-depth explanatory interviews with therapists, to investigate how they related to the intervention content and protocol format. In stage four, we responded to these qualitative themes through protocol modification and tailoring the focus of our therapist training programme.

### Assumptions

We adopted pragmatism as the underlying research paradigm: we approached our study objective from a pluralistic perspective, combined deductive and inductive modes of reasoning, and allowed for a singular view and multiple views of reality in interpreting our findings [12].

### Qualitative interviews: participants and recruitment

To reflect the feasibility study’s proposed population [9] and account for factors deemed potentially relevant in forming views of Morita therapy [10], we purposively sampled participants aged ≥ 18 with self-reported experience of depression, whether current or historic, and a range of previous therapy experience (potential patient sub-group) and therapists trained in complex psychological interventions such as CBT (therapist sub-group).

We recruited potential patients by email circulation to our research centre’s former participants who had consented to such contact and therapists by email circulation to current or former therapists in our centre.

### Procedure

Interviews were held at University of Exeter premises or the participant’s home and lasted between 45 and 130 min. Interviews combined qualitative techniques with those of cognitive interviewing [11], a method widely used when seeking an understanding of the cognitive processes involved in task completion [13] and recommended to capture participants’ immediate reactions to each intervention element [14].

#### Stage one

Interviews explored perceptions of Morita therapy in principle and practice. Prior to interview, we emailed participants a written summary of core Morita therapy principles on which to provide feedback. In line with prior research investigating novel interventions [15, 16], we then employed the vignette method to elicit views and understandings of the approach in practice, playing five audio-recording clips of the counselling-based modal model ranging from 3 to 5 min and each capturing a core element of the approach. We employed a variation of the think aloud technique [11], inviting participants to voice their thoughts during or after each vignette, according to their preference. At the end of each vignette, we used the open question ‘what are your thoughts on that?’ to allow flexibility and enable us to capture spontaneous responses [14].

Our topic guide was based on Morita therapy literature, the vignettes’ content and prior research addressing similar questions [16]. We included focussed questions to ensure discussion of each intervention element [14] as well as probing further into individual responses to investigate meanings, both exploring views on our pre-defined topics of interest and eliciting participants’ own themes [17]. Furthermore, we engaged in hypothesis testing as deemed appropriate, exploring the value of alternative explanations of concepts when misunderstanding of the vignettes was indicated.

#### Qualitative data analysis

Interviews were recorded, transcribed verbatim, managed within NVivo10 [18] and analysed using Framework analysis to allow for both inductive and deductive approaches [19], a method suitable for both data collected via cognitive interviewing [20] and health services research [21].

We used a combination of two approaches, namely Framework analysis and constant comparative analysis to analyse the data. Familiarisation with the data was achieved through producing and reading transcripts. We developed a thematic framework during preliminary analysis and subsequently as batches of transcripts were analysed, iteratively combining our topic guide with the overall narratives in context. Using this framework, we coded transcripts at the individual level and analysed them thematically across the whole dataset as well as in the context of each interview using a constant comparison approach [22], whereby each piece of data (e.g. one statement or theme) was compared with others for similarities and differences [23]. We thus formulated explanations, explored negative cases and provided explanations of variance [24]; ensuring perspectives which diverged from dominant themes were not overlooked [25]. To identify any sub-group differences, we undertook stage one analysis for potential patients first and subsequently for therapists. Given the resulting convergence of views within similar thematic frameworks, we developed analytic matrices [23] including all participants, allowing within and across case analyses, the exploration of relationships between themes and further refinement of themes through author discussions.

#### Stage two

In developing the draft protocol, we reviewed the English-language literature on the practice of Morita therapy to guide us in implementing the approach, most notably, Morita et al. 1998 [5]; Ogawa 2013 [6]; Nakamura et al. 2010 [7]; Ishiyama 2011 [26]; Ogawa 2007 [27]; LeVine 1993 [28]; LeVine, in press [29]; and personal communications: Minami, M. Through this process, we ensured adherence to the fundamental, defining features of Morita therapy (Table 1), considered akin to ‘guiding principles’ [14] which were essential to include in our protocol and formed the basis of the intervention.

**Table 1:** Key principles and practices of Morita therapy

	Term	Definition
Key principles	Natural world	Morita therapy conceptualises unpleasant thoughts and emotions as part of the natural ecology of the human experience. It draws upon the natural world, the place of humans within it, to emphasise that symptoms are not subject to the patient’s control and will naturally pass with time.
	Acceptance and allowance	All emotions and thoughts are accepted as they are. Attempts to control or resist symptoms are considered to exacerbate them; therapists thus help patients to move away from symptom preoccupation and combat and towards acceptance and a focus on action. Thus, the objectives of therapy are to shift attention and perspective, rather than controlling or ‘fixing’ symptoms.
	Rest	Morita therapy seeks to potentiate patients’ natural healing capacities, in contrast to resisting and exacerbating symptoms. Patients sit with their thoughts and emotions as they are, to learn how they naturally ebb and flow with time if attempts to control or remove them are not made and to build a natural desire to take action.
	Action-taking *with* symptoms	Patients learn to undertake purposeful and necessary action, with or without their symptoms. Morita therapy thus aims to improve everyday functioning in spite of symptoms, with symptoms reducing as a by-product of moving from a mood-oriented to a purpose-oriented and action-based lifestyle.
Key practices	Positive reinterpretation technique	Therapists ‘positively reinterpret’ symptoms as desires by seeing these as two sides of the same coin. For example, in Morita therapy, social anxiety represents a desire to be accepted by others. This technique aids acceptance of symptoms as natural and inevitable.
	Normalisation technique	Therapists label thoughts and emotions as ‘unpleasant’ and ‘pleasant’ but not ‘good’ or ‘bad’. They emphasise that all emotions are natural, or normal, and will ebb and flow on their own so long as attempts are not made to control or resist them.
	Fumon (inattention to symptoms)	Therapists, in an effort to shift patients’ attention away from symptom preoccupation and combat, will not focus on discussion or analysis of patients’ symptoms or their causes but rather will ‘steer’ the conversation towards action-taking and the external environment.
	Diaries	Patients complete daily diaries on which therapists provide comments which facilitate an acceptance of internal states and refocus attention on action and the external environment.
	Four-phased model	In traditional inpatient Morita therapy, rest and action-taking are structured within four phases: (1) complete bed rest; (2) light repetitive activities; (3) more challenging activities; and (4) social reintegration. The process is understood to aid experiential acceptance of the natural ebb and flow of thoughts and emotions, to re-orientate patients in nature and to refocus attention from internal to external states.

In response to our stage one findings, we also developed recommendations for optimising elements of Morita therapy for which multiple options were available in the literature and selected from the literature the delivery options considered most likely to address the issues raised. In addition, we included in the protocol specific stage one interview findings to address concerns and confusions, stress potentially valuable features and guide therapists in applying techniques.

#### Stage three

To review the draft protocol, we repeated interviews with the therapists from stage one, to enable them to reflect on the development of the approach and how well the protocol addressed their previous issues, plus an additional therapist recruited in the manner described, to capture the views of a therapist naïve to Morita therapy. Having emailed the protocol to therapists to read prior to their interview, we discussed their thoughts on the protocol and, to elicit views on all components, reviewed each protocol section in turn. Our topic guide was based on the draft protocol and stage one findings, with a focus on the extent of understanding obtained from the protocol, operationalising the therapy, protocol usability and accessibility, and areas on which to focus training.

#### Stage four

In amending the protocol in response to stage three, we re-referred to the Morita therapy literature to seek further guidance and ensure changes were grounded in the treatment’s fundamental features. Stage three findings also enabled us to tailor our therapist training programme by highlighting key issues and content to focus on.

## Results

We interviewed ten potential patients. All reported experience of depression; six had experience of psychotherapy and four did not (Table 2). The majority were female (*n* = 8, 80%); ages ranged from 22 to 63 years. We interviewed four therapists in stage one and five in stage three. All were trained in CBT and a mixture of other treatments such as behavioural activation; ages ranged from 43 to 63 years.

**Table 2:** Participant characteristics

Characteristics	Potential patients (*n* = 10)		Therapists (stage 1) (*n* = 4)		Therapists (stage 3) (*n* = 5)	
	*n*	%	*n*	%	*n*	%
Sex						
Male	2	20	2	50	2	40
Female	8	80	2	50	3	60
Age (in years)						
18–30	2	20	0	0	0	0
30–50	4	40	2	50	3	60
50–70	4	40	2	50	2	40
Nationality						
British	10	100	4	100	5	100
Highest level of education						
< A-levels	1	10	0	0	0	0
A-levels	2	20	0	0	0	0
University degree	5	50	1	25	1	20
Post-graduate diploma	0		1	25	2	40
Post-graduate degree	1	10	1	25	1	20
Doctoral degree	1	10	1	25	1	20
Mental health problem						
Depression	10	100	N/A		N/A	
Anxiety	8	80	N/A		N/A	
Previous therapy experience						
None	4	40	N/A		N/A	
Cognitive behavioural therapy	4	40	N/A		N/A	
Mindfulness-based cognitive therapy	3	30	N/A		N/A	
Behavioural activation	1	10	N/A		N/A	
Interpersonal psychotherapy	1	10	N/A		N/A	
Area(s) of clinical training						
Cognitive behavioural therapy	N/A		4	100	5	100
Behavioural activation	N/A		4	100	4	80
Eye movement desensitisation and reprocessing	N/A		1	25	2	40
Interpersonal psychotherapy	N/A		1	25	1	20
Dialectical behaviour therapy	N/A		1	25	1	20

*N/A* not applicable

### Stage one

**Fig. 1:**
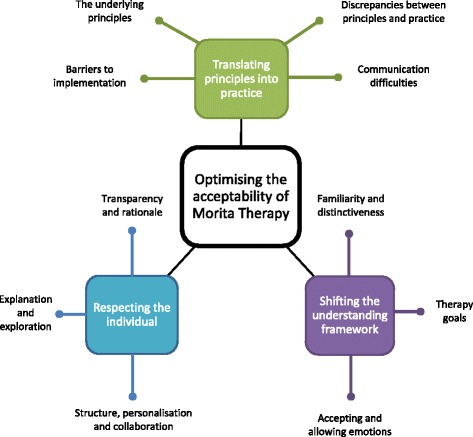
Stage one themes and constituent themes

Participants’ perspectives could be understood within three key themes: translating principles into practice, respecting the individual and shifting the understanding framework. Each key theme encompassed a number of constituent themes (Fig. 1: stage one themes and constituent themes).

In accordance with the objective of this paper to demonstrate the development of our protocol rather than presenting qualitative findings, we provide an exemplar of coded data for theme 1 (Table 3) to illustrate our analytical process, as opposed to including participant data for each theme.

**Table 3:** Exemplar of coded data: stage one theme one (translating principles into practice)

Constituent theme and elements	Participant responses
The underlying principles	
Learning to live with symptoms	‘I like that it’s about acceptance and accepting um the bad feelings you have rather than um fighting them all the time…yeah sort of living in spite of rather than trying to get rid of um, because it doesn’t work…it’s realistic.’ (Grace, potential patient)
Connecting to the natural world	‘That greater sense of being one with it all… I think that’s a very positive thing because it diffuses one’s own emotion…it puts what you are going through in context and that’s what this seemed to me in a way, um rather than being the centre of our universe as it were, we are part of it.’ (Claire, potential patient)
Viewing all emotions as natural phenomena	‘It’s a compassionate way of looking at yourself and what you’ve experienced as opposed to you shouldn’t be feeling like this.’ (Nicola, potential patient)
The vicious cycle of symptom aggravation	‘It does get into a cycle…you always tend to lean towards the, it, it almost feels easier to feel sad…and you do generally go over and over and over the unpleasant things.’ (Sarah, potential patient)
Rest	‘Giving yourself a bit of space…healing space, because I don’t always think there’s that in other kinds of therapies, there’s not that kind of re-charging space, um yeah, that’s nice.’ (Grace, potential patient)
Discrepancies between principles and practice	
Connection to the natural world	‘I liked the nature thing, but I didn’t hear that brought in.’ (Beth, potential patient)
Rest	‘I suppose what I construed from what I read is it’s more like actually if you don’t feel able then rest should be the mainstay of what you’re doing, rather than an hour in your day or a few minutes in your marathon…so I, yeah, I guess I feel kind of slightly less clear about the use of that sort of natural healing.’ (Hayley, therapist)
Resulting confusion	‘I don’t think that that [vignettes] matched this [summary of principles] at all, um really, so I’m going away from this…still wondering what Morita Therapy is.’ (Estelle, potential patient)
Communication difficulties	
Confusion in positive reinterpretation	‘My question to him would be if they’re flip sides then are they equal, so am I supposed to be worrying and enjoying something equal at the same time because I would disagree with that…I would say most of the time you should be looking at the positive and focusing on that…not you should be half worrying and half doing this.’ (Beth, potential patient)
	‘I remember somebody saying to me once nothing is either good or bad, it’s the way we react to it….somebody could get that impression…What I was going through with my parents…I’d be very interested to see how anybody could reframe for me in an acceptable way.’ (Claire, potential patient)
Barriers to implementation	
Diaries	‘I’ve always struggled with er sort of self-reflection in terms of writing… I think sometimes if it’s been a bad day, it kind of just all comes out and then I read it the next day and I just, it just looks like a load of rubbish… That’s, that’s the one thing that puts me off about doing it.’ (Mark, potential patient)
Action-taking	‘I find my depression and anxiety um quite paralysing, so saying about be anxious but get on with doing something, I find that I can’t.’ (David, potential patient)
Rest	‘Actually just saying hey just rest, I don’t find that very helpful because I need some order and structure and I think okay if I’m gonna rest at this point, who’s gonna clean the fish tank out, who’s gonna cook dinner, what do I do.’ (Sarah, potential patient)
Balancing action-taking and rest	‘Um dealing a little bit with this like paradox with action and also inaction, which is new… What are the parameters of rest, how is it structured…I’d like a little bit more structure around once you got to action.’ (Paul, therapist)

Note: Names changed to pseudonyms to protect confidentiality

*Translating principles into practice* illustrates participants’ responses to the written therapy principles and how these relate to the practice of therapy as demonstrated within the vignettes.

Generally, the principles of Morita therapy resonated positively. However, there was a lack of apparent translation of these into the vignettes and a sense of unmet expectations in practice. Of particular note was an absence of reference to the natural world and confusion caused by the presentation of ‘rest’. This perpetuated a lack of clarity regarding the purpose of rest and the treatment overall. Participants also demonstrated misunderstanding of messages conveyed in the vignettes, especially ‘positive reinterpretation’ (Table 1), indicating a need for increased clarity and specificity. Participants, whilst acknowledging the value of features such as diaries, rest and action-taking, also noted challenges around committing to these in practice.

*Respecting the individual* illustrates the extent to which Morita therapy was considered to be a well explained, individualised and collaborative approach.

The therapy process and intended outcomes were not considered clear from the vignettes, with mixed views on the acceptability of this: those with therapy experience generally expressed a need for full disclosure of rationale. Participants also expressed preferences for increased collaboration, such as seeking patient feedback, and more in-depth and personalised exploration and explanation of patients’ individual experiences and difficulties, particularly in relation to the normalisation technique (Table 1).

*Shifting the understanding framework* reflects how distinctive Morita therapy was considered to be and the extent to which it met participants’ expectations of effective therapy.

Overall, therapists acknowledged Morita therapy as a novel approach with a distinctive philosophical framework. Potential patients were less likely to note this, tending to interpret Morita therapy through the lens of other treatments and attempting to ‘fit’ the approach to those, generating some inaccurate assumptions. Potential patients also expressed tension between accepting unpleasant emotions, as per the premise of Morita therapy, and seeking techniques to change them. Thus, despite positive views of the holistic approach towards living well *with* symptoms, participants struggled to adopt this approach in considering the value of the overall therapy. Potential patients (especially those with therapy experience) focused more narrowly on mood-orientated goals, interpreting the features of therapy only as possible means of achieving the end of symptom reduction. However, therapists and treatment naïve potential patients often valued how the therapy provided insight, shifted attention, and potentially changed one’s relationship to emotions without changing emotions themselves.

#### In summary

Our findings indicated that the core Morita therapy features were largely acceptable to participants, albeit with potential for improvement in how these are conveyed and structured in order to enhance the relevance, comprehensibility and appeal of the approach.

### Stage two

The Morita therapy literature demonstrated a range of potential methods for implementing, communicating and structuring the key features of Morita therapy, which were thus open to tailoring to the target population. Overall, the delivery options fall along a spectrum (personal communications: Minami, M) from prescriptive inpatient settings adhering to a four-phased experiential structure [5] to exploratory outpatient counselling methods with no such structure, such as the active counselling method [26] and modal model (personal communications: Minami, M), which apply and extend the guidelines for outpatient Morita therapy [7].

In selecting from these options during the development of our therapy protocol, we shifted our approach along the spectrum of treatment modes from the counselling-based method alone (as presented in the vignettes) towards the traditional experiential four-phased approach (Table 1). This addressed our stage one findings by strengthening the core components and overarching structure of the approach, reinforcing the process and purpose of therapy, and balancing otherwise somewhat paradoxical features such as rest and action-taking within a clearly defined structure.

To address the challenges highlighted by participants in relation to completing diaries and rest, we stressed the need for an individualised, flexible and reassuring approach to identifying patients’ concerns and capabilities. As indicated necessary by our qualitative results, we stressed the importance of delivering therapy in a personalised, collaborative and well explained manner. We provided clear guidance and warning points on implementing techniques such as positive reinterpretation and normalisation, to address the misunderstandings and concerns raised.

One key qualitative message was that care would be required in explaining the purpose of therapy and managing the ways in which it may differ from patients’ preconceptions and prior experiences. Thus, one protocol inclusion is a managing patients’ expectations section, intended to facilitate a shift in patients’ understanding frameworks from the beginning of treatment, and ensure provision of the desired level of transparency and rationale.

We have selected the rest phase to illustrate how we developed the protocol (Table 4) and Additional file 1 provides further details of the ways in which our qualitative themes were refined into recommendations and subsequently informed our protocol development.

**Table 4:** Exemplar of therapy protocol development: stage two (the rest phase)

Stage two: development of the draft protocol	We developed each of the four phases of Morita therapy into separate sections following our decision, on the basis of our qualitative findings, to structure the therapy according to this model
	To produce the rest phase section, we amalgamated the Morita therapy literature on engaging in rest to provide an overview and general guidance for preparing patients for rest (personal communications: Minami, M), specific instructions for developing an appropriate schedule and environment for rest ([5, 7, 27, 28, 30]; personal communications: Minami, M) and guidance on the indicators of progress during rest (personal communications: Minami, M)
	In incorporating our qualitative findings, we included potential patients’ feedback on their fears of and barriers to rest
	To guide therapists in addressing such issues, we provided guidance on stressing the importance of and rationale for rest, drawing on physical health and natural metaphors in explaining rest, and exploring and tackling feelings of guilt around taking rest, as suggested valuable from our qualitative themes
	In order to address the misinterpretations of the meaning and nature of rest encountered in our interviews, we provided warning points for these potential misinterpretations as well as clear guidance on managing patients’ expectations of the purpose and likely experience of rest
	We included specific instructions for the conditions for taking rest to further assuage doubts around the meaning of rest in Morita therapy

### Stage three

**Fig. 2:**
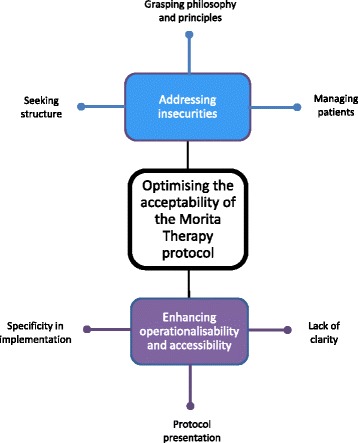
Stage three themes and constituent themes

Therapists’ perspectives in the context of the draft protocol could be understood within two key themes: addressing insecurities and enhancing operationalisability and accessibility. Each key theme encompassed a number of constituent themes (Fig. 2: stage three themes and constituent themes).

*Addressing insecurities* illustrates the concerns therapists expressed around orientating to and delivering therapy.

Therapists noted that the protocol provided much understanding and addressed many issues previously raised. However, considering the approach novel and only deliverable from a thorough grounding in the principles, they demonstrated a lack of confidence and noted the need to emphasise key principles to adhere to. They sought to simplify the approach, understand it in terms of more familiar therapies and, despite acknowledgement of the potential incompatibility with Morita therapy, enhance its structure through clearly defined timeframes and content outlines for each therapy session.

Although acknowledging the usefulness of the guidance on managing expectations, therapists expressed trepidation around this, stressing the importance of role playing during training, seeking examples of managing typical patient responses and desiring a pre-treatment patient summary sheet. Therapists often noted concerns around implementing rest and doubts as to the rationale for this. Accordingly, they desired more clearly defined instructions for instigating rest and flexibility around engagement with rest dependent on patient presentation and preference.

*Enhancing operationalisability and accessibility* illustrates therapists’ suggestions for improving protocol presentation and areas in which they felt more guidance, clarity or specificity was required.

Overall, therapists considered the protocol thorough, understandable and user-friendly. However, further clarity was required, especially in balancing features such as direction with collaboration, and Fumon (inattention to symptoms, Table 1) with empathy. Therapists appreciated the current inclusion of stage one interview findings and desired more verbatim clinical illustrations to guide them in implementing techniques, choosing appropriate activities and commenting on diaries. Noting the subtlety of the indicators of therapeutic progress, therapists suggested value in delineating these clearly in line with treatment objectives and illustrative examples.

Therapists queried whether they should use Japanese terms, desired a glossary of these, and noted the lack of explicit specification of the number and spacing of therapy sessions. Furthermore, they considered the protocol somewhat difficult to digest, indicating the value of additional summaries and crib sheets, and of further compartmentalisation through bullet points and highlighting of key interview findings.

#### Summary

Our findings indicated that the protocol required improvements in format to enhance ease of use; additional guidance, specificity or clarity to address the issues raised.

### Stage four

To optimise the protocol in response to our stage three findings, we added verbatim illustrations where available from the literature and, to provide precision in assessing indicators of progress, re-structured the protocol to link these explicitly to key objectives and examples. To adhere to Morita therapy practice, we did not provide session content outlines and clarified that all patients should partake in rest. We added guidance on balancing direction with collaboration, specified the number and spacing of therapy sessions, added a glossary of Japanese terms noting no requirement to use these during treatment and clarified the types of/conditions for patient activities.

In amending the protocol presentation, we added summaries and concise guidance; deconstructed guidance into bullet points and tables; delineated key features, tips, techniques and warning points in boxes; and incorporated colour and bold text to enhance accessibility. We developed one-page summary sheets to simplify key concepts, techniques and phases of treatment alongside their purpose, conditions and indicators of progress. We developed a pre-treatment patient handout, to begin expectation management at the earliest opportunity.

We have illustrated our continued development of the therapy protocol using the rest phase section (Table 5).

**Table 5:** Exemplar of therapy protocol development: stage four (the rest phase)

Stage four: modification of the draft protocol	• We edited the rest phase section to ensure the guidance was concise and increase the use of bullet points
	• We deconstructed key features of rest (analogies to physical health, tackling guilt), tips for explaining rest (using metaphors to describe the rationale, experience and nature of rest), techniques for preparing for rest (silent sitting) and warning points (e.g. potential misinterpretations of the meaning of rest) into boxes of different colour to aid ease of use
	• We delineated the indicators of progress in a table relating each to a conceptual objective, means of assessment and verbatim examples of patients demonstrating the indicator as identified from a further review of the literature (personal communications: Minami, M)
	• We developed a summary sheet for negotiating and engaging in the rest phase (guidelines, purposes and indicators of progress) to provide simplified and accessible key guidance to refer to during a therapy session
	• The pre-treatment patient handout was made suitable to be provided to patients’ significant others when embarking on the rest phase, to provide additional support for patients during this phase and thus ease therapists’ concerns in this area
	• As well as clarifying the instructions to provide to patients entering the rest phase, we clarified that all patients, regardless of presentation, should engage in as much rest as possible, in order to address confusion around assessing this and stress that, in the event of patients’ reluctance to engage in rest, reiterating the importance of and rationale for rest should be prioritised over missing this phase
	• Thus, whilst acknowledging and addressing the challenges of the rest phase for both patients and therapists, we adhered to the literature which deems rest, or at least silent sitting, fundamental to Morita therapy [5, 7, 28]

In tailoring our therapist training programme, we maintained a focus on grounding in the key principles to enhance therapists’ confidence. We focused role plays on implementing and balancing therapeutic techniques, managing patient expectations and responses, delivering rationale, guiding patients through treatment phases and identifying suitable and personalised activities for patients. In the absence of diary illustrations in the literature, we incorporated commenting on mock diaries and discussions around key principles to adhere to in doing so.

## Discussion

The overall aim of this paper is to showcase best practice in intervention development through describing a systematic, iterative, person-based approach to optimising intervention feasibility and acceptability, illustrated by its application to the development of the UK Morita therapy outpatient protocol. We have presented examples of how qualitative findings were integrated with Morita therapy literature in order to sensitively adapt the intervention across cultures whilst carefully ensuring adherence to its fundamental features.

Our first stage utilised in-depth exploratory qualitative interviews, drawing on techniques of cognitive interviewing [11] and vignettes of therapy delivery in order to explore potential patients’ and therapists’ perspectives of Morita therapy in principle and practice. Our findings demonstrated that the core features were acceptable for participants whilst highlighting the potential for improvement in their implementation, for which scope for tailoring the approach was available. Secondly, we synthesised the Morita therapy literature whilst accounting for and incorporating our qualitative findings and resulting recommendations for optimising the intervention.

Our third stage utilised in-depth explanatory repeat qualitative interviews with therapists, aided by the draft protocol itself, to investigate responses to the resulting intervention content, reflect on the intervention development and explore views on protocol presentation. Our findings indicated that the draft protocol addressed many of the issues previously raised, providing comprehensive and understandable guidance, whilst highlighting requirements for further guidance and improved accessibility. Finally, we re-examined the Morita therapy literature to assist us in addressing these issues, improving the protocol presentation and tailoring the focus of our therapist training programme. As such, we developed a therapy protocol and training programme which were fit for purpose in proceeding to a UK-based Morita therapy feasibility study.

### Limitations

HVRS, who conducted all interviews, was also involved in the protocol development process. Thus, particularly in the repeat interviews, although questions were posed to deliberately elicit negative views, participants may have been reluctant to express criticism of the draft protocol. However, participants did freely indicate ways in which the protocol was currently confusing, insufficient or inaccessible. In addition, in the absence of vignettes demonstrating a variety of treatment models, we were unable to elicit participants’ views on all available options so as to elect a favoured approach and instead used their feedback on the modal model to guide us in positioning our version of therapy along the available spectrum. Furthermore, although our sample was diverse in age, gender and therapy experience and may well represent those most likely to be interested in receiving Morita therapy, certain sectors of the UK population such as ethnic minority groups were clearly underrepresented.

## Conclusions

This process has enabled us to proceed to the feasibility study [9] with a therapy protocol which, whilst adhering to the essence of Morita therapy, has enhanced acceptability and feasibility for a UK population, thus maximising the likelihood of a successful outcome in this study [10]. During the feasibility study we are continuing our assessment of intervention acceptability through post-treatment qualitative interviews and a mixed method analysis exploring the relationship between participants’ views, therapist fidelity to the protocol and patient adherence to treatment. Further intervention modifications may well be suggested by such findings, enabling us to continue this iterative process of optimising the approach for a UK population in preparing for the first large-scale evaluation of Morita therapy in the UK.

We showcase best practice in intervention development by transparently illustrating a systematic approach which prioritises the perspectives of those who will both deliver and receive the intervention and integrates user feedback with literature synthesis in an iterative, thorough and replicable design. In line with the person-based approach to enhancing the acceptability and feasibility of interventions, we have thus grounded our development process in ‘a sensitive awareness of the perspective and lives of the people who will use [it]’ ([10] p.1), utilising both written materials and vignettes of therapy delivery in order to elicit views on every intervention element and repeating interviews to check acceptability and accessibility. Without undertaking this study, we would not have understood the expectations, understandings and needs of stakeholders and the ways in which these may shape their delivery of and engagement with the intervention. Whilst this was key in the specific cross-cultural adaptation of a novel intervention, we present a generalisable approach to optimising interventions which is likely to be relevant and interesting to others in both the development and evaluation of complex interventions.

## Additional file

Additional file 1: The use of stage one findings to inform stage two therapy protocol development. Table detailing the stage one themes, resulting recommendations for optimising Morita therapy for this population and ways in which such recommendations informed our development of the therapy protocol during stage two. (XLSX 16 kb)

## Abbreviations

CBT: Cognitive behavioural therapy
GAD: Generalised anxiety disorder
